# Association of glucose metabolism status and paraspinal muscle degeneration with chronic pain after endoscopic lumbar decompression: a multicentre retrospective study

**DOI:** 10.3389/fendo.2026.1867106

**Published:** 2026-05-29

**Authors:** Shihao Zhou, LingZhi Li, Zhenxian Qi, Junhao Sun, Tianluo Guo, Peiran Hu, Bin Yuan, Hengji Li, Guilan Gou, Zhanyin Li, Hongshun Zhao, Yajun Deng, Dazhi Yang, Jiancuo A.

**Affiliations:** 1 Graduate School of Qinghai University, Xining, China; 2 Department of Spine Surgery, Qinghai Red Cross Hospital, Xining, China; 3 Department of Pain Medicine, Affiliated Hospital of Hebei University, Baoding, China; 4 School of Medicine, Shenzhen University, Shenzhen, Guangdong, China; 5 Nanshan Hospital Affiliated to Shenzhen University, Shenzhen, Guangdong, China; 6 Department of Orthopedics, Xi’an Daxing Hospital, Xi’an, Shaanxi, China

**Keywords:** chronic low back pain, diabetes mellitus, lumbar disc herniation, paraspinal muscles, unilateral biportal endoscopy

## Abstract

**Objective:**

To investigate the associations of glucose metabolism status and paraspinal muscle degeneration with chronic low back pain (CLBP) after endoscopic lumbar decompression, and to evaluate the clinical significance of metabolic abnormalities, paraspinal muscle fat infiltration, and psoas muscle reserve in persistent postoperative pain.

**Methods:**

A total of 1,076 patients with lumbar disc herniation (LDH) who underwent unilateral biportal endoscopic (UBE) decompression at three medical centres between January 2021, and December 2024 were retrospectively included. Based on follow-up outcomes, patients were classified into CLBP and non-CLBP groups. Preoperative imaging parameters included multifidus fatty infiltration (MF FI), erector spinae fatty infiltration (ES FI), and the psoas muscle index (PMI). Univariable and multivariable logistic regression analyses were performed to identify factors independently associated with postoperative CLBP. Restricted cubic spline (RCS) analysis was used to assess potential nonlinear associations between key variables and postoperative CLBP risk.

**Results:**

Postoperative CLBP developed in 328 patients. Compared with patients in the non-CLBP group, those in the CLBP group were older, had higher HbA1c levels, and had lower PMI values. MF FI and ES FI were also higher in the CLBP group (both P < 0.001). Multivariable logistic regression analysis showed that age (OR = 1.07), HbA1c (OR = 1.16), MF FI (OR = 1.10), and ES FI (OR = 1.13) were independently associated with an increased risk of postoperative CLBP, whereas PMI was protective (OR = 0.71; all P < 0.001). RCS analysis indicated nonlinear associations of HbA1c, age, and MF FI with postoperative CLBP risk, whereas ES FI and PMI showed approximately linear associations.

**Conclusion:**

The risk of CLBP after endoscopic lumbar decompression was associated not only with local decompression but also with preoperative glucose metabolism status, paraspinal muscle fat infiltration, and overall muscle reserve. Metabolic abnormalities, degeneration of the local stabilising system, and reduced muscle reserve may collectively contribute to persistent postoperative pain.

## 1 Introduction

Lumbar degenerative disease is a common cause of low back and leg pain and functional limitation in middle-aged and older adults. For patients who fail to respond to conservative treatment and show concordance between clinical symptoms and imaging findings, decompression surgery remains an important option for relieving neural compression and improving function. In recent years, endoscopic lumbar decompression has been increasingly used in clinical practice because it causes less tissue trauma and allows faster recovery (1, 2). However, some patients continue to experience postoperative pain, which may progress to a chronic pain state (3). Adequate radiographic decompression does not always result in satisfactory clinical outcomes. This finding suggests that postoperative pain may be influenced not only by the relief of local neural compression but also by multiple other factors (4). The paraspinal muscles constitute an important dynamic system that maintains lumbar stability and functional integrity. These muscles play a key role in segmental stability, load distribution, and postural control (5). Previous studies have shown that, compared with healthy individuals, patients with lumbar degenerative disease are more likely to have increased paraspinal muscle fatty infiltration, reduced muscle quality, and impaired muscle function. These changes have been associated with greater low back pain severity, functional disability, and reduced spinal stability (6, 7). Compared with muscle cross-sectional area alone, fatty infiltration and functional cross-sectional area better reflect the effective contractile component and functional status of muscle (8). In addition to the paraspinal muscles, the psoas major is also involved in trunk stability and load transfer. Standardized psoas indices are commonly used to evaluate overall muscle reserve. Previous evidence suggests that insufficient psoas muscle reserve may be associated with reduced physical capacity and unfavourable surgical outcomes. Therefore, psoas muscle reserve may be an important factor affecting postoperative pain recovery (9). Beyond local muscle factors, abnormal glucose metabolism has also been linked to inadequate pain relief and poor functional recovery after spinal surgery. The potential mechanisms may include chronic low-grade inflammation, microcirculatory dysfunction, impaired peripheral nerve function, and reduced tissue repair capacity (10). In addition, abnormal glucose metabolism may contribute to skeletal muscle degeneration (11). Clinically, poor glycaemic control is often accompanied by reduced muscle mass, increased fat deposition, and impaired physical performance. These findings suggest that poor postoperative recovery in these patients may be related not only to metabolic abnormalities but also to more pronounced paraspinal muscle degeneration and insufficient overall muscle reserve. Accordingly, glucose metabolic status and muscle imaging features may jointly contribute to chronic postoperative pain.

Current studies of chronic pain after endoscopic lumbar decompression have mainly focused on surgical technique, affected spinal level, and general clinical characteristics. However, the combined evaluation of glucose metabolic status and muscle imaging features has received relatively limited attention. In particular, few studies have systematically examined the associations among glucose metabolic status, paraspinal muscle fatty infiltration, psoas muscle reserve, and chronic postoperative pain. Therefore, patients undergoing endoscopic lumbar decompression were enrolled to analyse the associations between glucose metabolic status, paraspinal muscle imaging features, and chronic postoperative pain. The potential clinical value of these factors in preoperative risk assessment and precision perioperative management was further explored.

## 2 Materials and methods

### 2.1 Patient selection

**Figure 1:**
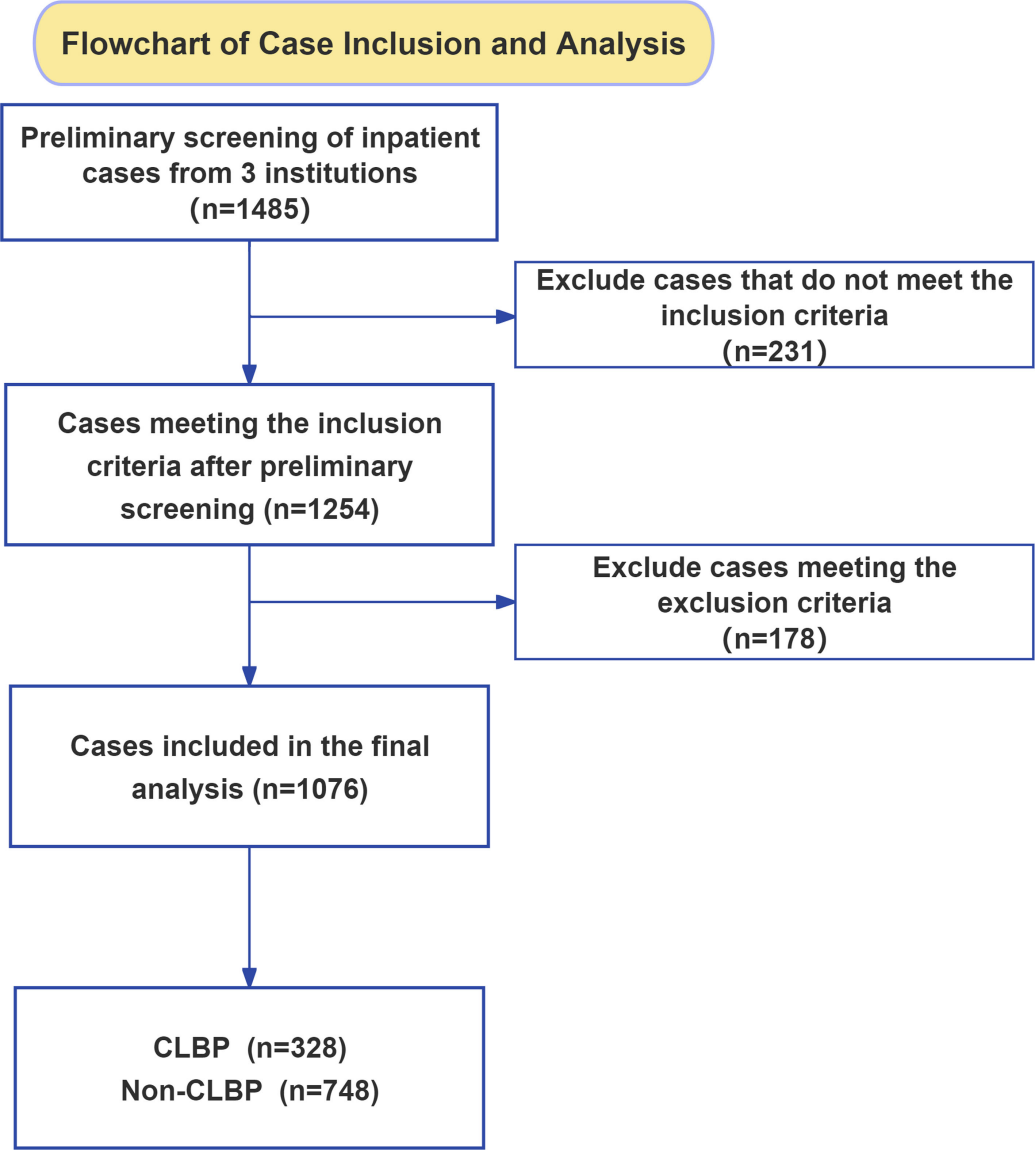
Flowchart of case inclusion and analysis.

This was a multicentre retrospective study. A total of 1,076 patients with lumbar disc herniation (LDH) who underwent unilateral biportal endoscopic (UBE) decompression between January 2021, and December 2024 were enrolled from Qinghai Red Cross Hospital, Xi’an Daxing Hospital, and Shenzhen Nanshan People’s Hospital. The inclusion criteria were: (1) single-level LDH, with or without sensory abnormalities in the corresponding nerve root distribution; (2) typical unilateral radicular pain, numbness, or lower-limb muscle weakness, with no significant improvement after at least 3 months of standardised conservative treatment; (3) agreement between clinical findings and imaging results; and (4) complete follow-up data. The exclusion criteria were: (1) revision surgery at the same segment; (2) coexisting spinal disorders that could substantially affect postoperative pain outcomes, including ankylosing spondylitis, spinal tumours, fractures, or tuberculosis; (3) incomplete imaging data; (4) instability of the target segment requiring fusion; (5) loss to follow-up or incomplete outcome data; (6) long-term use of analgesics before surgery; (7) documented severe psychological or psychiatric disorders that could interfere with pain assessment; and (8) postoperative pain attributable to definite organic causes, such as infection, dural tear, or recurrent disc herniation. The study flowchart is presented in **Figure 1**. All procedures were performed using a standardised surgical approach by three senior spine surgeons with extensive experience in minimally invasive techniques, thereby minimising procedural variability. All patients were followed up for more than 1 year after surgery. Postoperative chronic low back pain (CLBP) was defined as low back pain persisting for more than 6 months after surgery, with a visual analogue scale (VAS) score >4. Patients with identifiable organic causes of pain, such as infection, dural tear, or recurrent disc herniation, or severe psychological factors that could interfere with pain assessment, were excluded (12–17). According to these criteria, patients were classified into CLBP and non-CLBP groups. This study was led by Qinghai Red Cross Hospital and was approved by the ethics committees of all participating centres (approval numbers: KY-2025-154, LW2025-026, and KY-2025-073001). The study was conducted in accordance with the Declaration of Helsinki. To reduce heterogeneity, consistent control measures were applied during study design, data collection, and statistical analysis. Uniform inclusion and exclusion criteria, outcome definitions, and surgical procedures were adopted across all centres.

### 2.2 Imaging feature extraction

**Figure 2:**
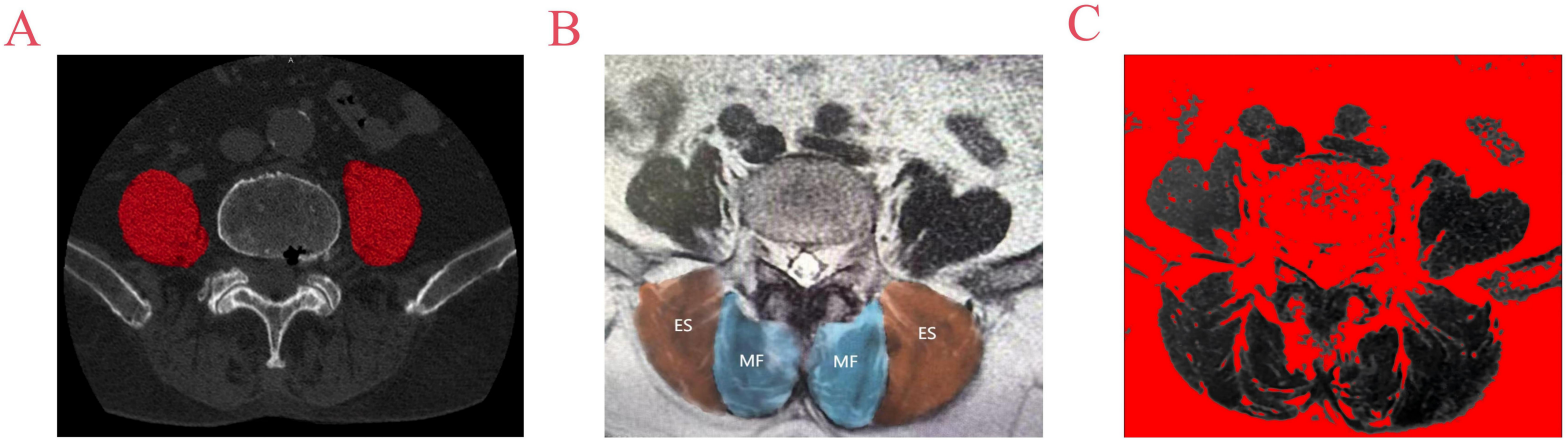
Imaging-based muscle measurements. **(A)** Manual segmentation of bilateral psoas muscles on axial CT at L4 (red) for CSA and PMI calculation. **(B)** ROI delineation of the paraspinal muscles on axial MRI at the inferior L4 endplate, including MF and ES. **(C)** Threshold-based segmentation in ImageJ to quantify FI; red mask indicates pixels meeting the preset threshold.

Psoas muscle parameters were extracted from CT images. At the L4 vertebral level, the bilateral psoas muscles were manually segmented using ImageJ with a standardised threshold of −29 to +150 Hounsfield units (HU). The cross-sectional area (CSA) was then measured (**Figure 2A**). The psoas muscle index (PMI) was calculated as the sum of the bilateral psoas CSA divided by height squared (cm²/m²).Paraspinal muscle parameters were obtained from MRI images. At the inferior endplate of L4, regions of interest (ROIs) for the multifidus and erector spinae muscles were manually delineated along the muscle boundaries. Surrounding adipose tissue was excluded as far as possible to ensure accurate representation of muscle morphology and size (**Figure 2B**). After segmentation, the CSA of each region was automatically calculated, and fat infiltration (FI) was assessed. The fat infiltration area was measured in ImageJ using a threshold-based method with a range of 90–255 on 8-bit greyscale images. Pixels within this range were displayed as a red mask, and the fat areas of the multifidus and erector spinae muscles were calculated accordingly (**Figure 2C**). The functional cross-sectional area (FCSA) was defined as the total CSA minus the fat area and was normalised by height squared (cm²/m²). The degree of fat infiltration was calculated as the percentage of fat area relative to the total muscle area. All imaging measurements were performed twice, and the mean values were used in subsequent analyses. All imaging data were independently evaluated by three experienced senior spine surgeons who were blinded to the clinical data and outcomes. All patient data were anonymised and coded before analysis, and no identifiable personal information was used throughout the study. To assess measurement consistency and reliability, data from 60 randomly selected patients were used for intraclass correlation coefficient (ICC) analysis. ICC values for both single and average measurements were calculated for each imaging parameter to evaluate data stability and reliability.

### 2.3 Statistical analysis

Statistical analyses were performed using SPSS version 27.0. Data normality was assessed using the Shapiro–Wilk test. Categorical variables are presented as frequencies and percentages (N (%)), whereas continuous variables are presented as the mean ± standard deviation. Normally distributed continuous variables were compared using Student’s t test, whereas non-normally distributed variables were compared using the Mann–Whitney U test. Categorical variables were compared using the chi-square test. A two-sided P value of <0.05 was considered statistically significant. Univariable and multivariable logistic regression analyses were performed to identify independent risk factors for CLBP. Restricted cubic spline (RCS) models were used to further explore and visualize potential nonlinear associations between key continuous variables and the risk of postoperative CLBP. The RCS models were fitted with three knots, with knot locations automatically determined according to the distribution of each variable. This approach was applied to improve model stability and reduce the risk of overfitting. Overall and nonlinear associations were considered statistically significant at P < 0.05. HbA1c was included as a continuous variable in both the logistic regression and RCS analyses and was not categorized according to predefined clinical thresholds.

## 3 Results

### 3.1 Interobserver reliability assessed by ICC

The reproducibility and reliability of all imaging parameters were assessed using ICC analysis (**Table 1**). Based on data from 60 randomly selected patients, all imaging measurements showed good consistency. The ICC values for PMI, MF FI, and ES FI were approximately 0.84. The ICC values for MF FCSA and ES FCSA were slightly higher, at 0.85 and 0.87, respectively. These findings indicate good reproducibility and stability across evaluators. Overall, all imaging parameters showed strong agreement across raters and repeated measurements, supporting the reliability of the imaging assessment methods used in this study.

**Table 1:** Reliability analysis of imaging-derived parameters.

Parameter	ICC value	95% CI		t	*P*
PMI	0.844	0.803	0.878	17.941	<0.001
MF FI	0.845	0.803	0.879	18.277	<0.001
ES FI	0.843	0.804	0.876	17.180	<0.001
MF FCSA	0.853	0.817	0.885	18.868	<0.001
ES FCSA	0.868	0.835	0.896	20.947	<0.001

### 3.2 Comparison of Baseline Characteristics and Clinical Features Between the Two Groups

A total of 1,076 patients were included in the study, comprising 748 in the non-CLBP group and 328 in the CLBP group. Compared with patients in the non-CLBP group, those in the CLBP group were older, had higher HbA1c levels, and had lower PMI values. The CLBP group also showed greater fatty infiltration of the multifidus and erector spinae muscles, as reflected by higher MF FI and ES FI values (both P < 0.001).The distribution of herniated disc levels also differed between the two groups (P = 0.039), with a higher proportion of L4/5 involvement in the CLBP group. No significant differences were observed between the groups in sex, body mass index, alcohol consumption, smoking status, MF FCSA/m², ES FCSA/m², operative time, or disc herniation type (all P > 0.05; **Table 2**).

**Table 2:** Baseline characteristics of patients in the CLBP and non-CLBP groups.

Variables	NO CLBP (n = 748)	CLBP (n = 328)	Statistic	*P*
Age,years	67.42 ± 10.55	73.39 ± 10.15	t=-8.65	<0.001
Gender, n(%)			χ²=1.99	0.158
Male	393 (52.54)	157 (47.87)		
Female	355 (47.46)	171 (52.13)		
BMI,kg/m²	25.94 ± 5.96	25.82 ± 5.76	t=0.31	0.759
Drink, n(%)	459 (61.36)	198 (60.37)	χ²=0.10	0.757
Smoke, n(%)	397 (53.07)	170 (51.83)	χ²=0.14	0.706
HbA1c, %	8.13 ± 1.61	8.58 ± 1.88	t=-3.72	<0.001
PMI, cm²/m²	5.17 ± 1.48	4.47 ± 1.36	t=7.67	<0.001
MF fCSA/m^2^,cm²/m²	3.32 (2.92, 3.73)	3.29 (2.90, 3.66)	Z=-1.18	0.238
ES fCSA/m^2^,cm²/m²	10.48 (9.15, 12.20)	10.56 (9.19, 12.36)	Z=-0.85	0.395
MF FI,%	20.37 ± 4.64	22.45 ± 5.30	t=-6.14	<0.001
ES FI,%	16.19 ± 3.83	18.26 ± 4.00	t=-8.05	<0.001
Operation time,min	104.55 ± 4.90	104.69 ± 4.89	t=-0.44	0.658
Herniation type, n(%)			χ²=2.59	0.108
Central	329 (43.98)	127 (38.72)		
Lateral	419 (56.02)	201 (61.28)		
Level of herniated disc, n(%)			χ²=6.51	0.039
L3/4	87 (11.63)	28 (8.54)		
L4/5	460 (61.50)	228 (69.51)		
L5/S1	201 (26.87)	72 (21.95)		

### 3.3 Univariate and multivariable logistic regression analyses

The results of the univariable and multivariable logistic regression analyses are shown in **Tables 3**, **4**. After adjustment for age, sex, BMI, smoking history, alcohol consumption, surgery-related factors, and imaging parameters, age, HbA1c, PMI, MF FI, and ES FI were independently associated with postoperative CLBP. Advanced age was identified as an independent risk factor for postoperative CLBP (OR = 1.07, 95% CI: 1.05–1.09, P < 0.001). Elevated HbA1c was significantly associated with an increased risk of postoperative CLBP (OR = 1.16, 95% CI: 1.08–1.25, P < 0.001). Each 1% increase in HbA1c was associated with an approximately 16% increase in postoperative CLBP risk. In contrast, PMI was identified as a protective factor (OR = 0.70, 95% CI: 0.64–0.77, P < 0.001), indicating that greater psoas muscle reserve was associated with a lower risk of postoperative CLBP. Each one-unit increase in PMI was associated with an approximately 30% reduction in postoperative CLBP risk. In addition, both MF FI (OR = 1.09, 95% CI: 1.06–1.12, P < 0.001) and ES FI (OR = 1.14, 95% CI: 1.10–1.18, P < 0.001) were significant risk factors. No significant associations were observed between postoperative CLBP and sex, BMI, smoking status, alcohol consumption, operative time, disc herniation type, or segmental distribution (all P > 0.05). After further model optimization using statistically significant variables, age, HbA1c, PMI, MF FI, and ES FI remained independently associated with postoperative CLBP. HbA1c (OR = 1.16, 95% CI: 1.07–1.26, P < 0.001), MF FI (OR = 1.10, 95% CI: 1.07–1.13, P < 0.001), and ES FI (OR = 1.13, 95% CI: 1.09–1.18, P < 0.001) remained significantly associated with an increased risk of postoperative CLBP, whereas PMI remained protective (OR = 0.71, 95% CI: 0.64–0.79, P < 0.001).

**Table 3:** Results of univariate logistic regression analysis.

Variables	β	S.E	OR (95%CI)	Z	*P*
Age	0.07	0.01	1.07 (1.05 ~ 1.09)	8.00	<0.001
Gender, n(%)					
Male			1.00		
Female	0.19	0.13	1.21 (0.93 ~ 1.56)	1.41	0.158
BMI	-0.00	0.01	1.00 (0.97 ~ 1.02)	-0.31	0.759
Drink, n(%)	-0.04	0.14	0.96 (0.74 ~ 1.25)	-0.31	0.757
Smoke, n(%)	-0.05	0.13	0.95 (0.73 ~ 1.23)	-0.38	0.706
HbA1c	0.15	0.04	1.16 (1.08 ~ 1.25)	3.88	<.001
PMI	-0.36	0.05	0.70 (0.64 ~ 0.77)	-7.08	<.001
MF fCSA	-0.17	0.14	0.84 (0.64 ~ 1.11)	-1.24	0.215
ES fCSA	0.03	0.04	1.03 (0.96 ~ 1.11)	0.89	0.372
MF FI	0.09	0.01	1.09 (1.06 ~ 1.12)	6.26	<0.001
ES FI	0.13	0.02	1.14 (1.10 ~ 1.18)	7.65	<0.001
Operation time	0.01	0.01	1.01 (0.98 ~ 1.03)	0.44	0.658
Herniation type, n(%)					
Central			1.00		
Lateral	0.22	0.14	1.24 (0.95 ~ 1.62)	1.61	0.108
Level of herniated disc, n(%)					
L3/4			1.00		
L4/5	0.43	0.23	1.54 (0.98 ~ 2.43)	1.86	0.063
L5/S1	0.11	0.26	1.11 (0.67 ~ 1.84)	0.42	0.677

**Table 4:** Results of multivariate logistic regression analysis.

Variables	β	S.E	OR (95%CI)	Z	*P*
Age	0.07	0.01	1.07 (1.05 ~ 1.09)	-10.13	<0.001
HbA1c	0.15	0.04	1.16 (1.07 ~ 1.26)	7.96	<0.001
PMI	-0.34	0.05	0.71 (0.64 ~ 0.79)	3.58	<0.001
MF FI	0.09	0.02	1.10 (1.07 ~ 1.13)	-6.28	<0.001
ES FI	0.13	0.02	1.13 (1.09 ~ 1.18)	6.22	<0.001

### 3.4 Nonlinear association analysis

**Figure 3:**
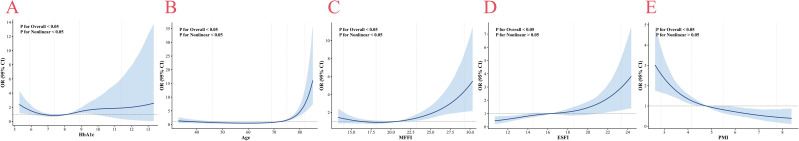
Nonlinear relationships between continuous variables and the risk of the outcome. **(A)** HbA1c; **(B)** age; **(C)** MFFI; **(D)** ESFI; **(E)** PMI. The solid lines represent ORs, and the shaded areas represent 95% CIs.

RCS analysis showed that HbA1c, Age, MF FI,ES FI, and PMI were associated with the risk of postoperative CLBP (all P < 0.05, **Figure 3**). Among these variables, HbA1c, age, and MF FI showed nonlinear associations with postoperative CLBP risk (all P < 0.05). In contrast, no clear nonlinear associations were observed for ES FI or PMI (both P > 0.05).HbA1c showed a nonlinear association with postoperative CLBP risk. The risk was relatively lower at intermediate HbA1c levels and increased progressively at higher levels. Age also showed a nonlinear association, with a more pronounced increase in risk in older patients. MF FI was positively and nonlinearly associated with postoperative CLBP. The risk increased gradually with greater fatty infiltration of the multifidus muscle and rose more sharply at higher FI levels. By comparison, ES FI showed an approximately linear positive association with postoperative CLBP, suggesting that greater fatty infiltration of the erector spinae muscle was associated with a higher risk. PMI showed an approximately linear negative association, suggesting that greater psoas muscle reserve was associated with a lower risk of postoperative CLBP. Overall, abnormal glucose metabolism, older age, increased paraspinal muscle fatty infiltration, and reduced psoas muscle reserve were associated with postoperative CLBP. Among these factors, HbA1c, age, and MF FI showed nonlinear effects on risk.

## 4 Discussion

This study showed that CLBP after endoscopic lumbar decompression was associated with several factors. In addition to local neural decompression, postoperative CLBP was associated with preoperative glucose metabolic status, paraspinal muscle degeneration, and overall muscle reserve. Multivariable logistic regression analysis showed that age, HbA1c, MF FI, and ES FI were independently associated with an increased risk of postoperative CLBP, whereas PMI was protective. These findings suggest that preoperative risk assessment should incorporate metabolic status and muscle imaging features in addition to structural lesions.

### 4.1 Relationship between paraspinal and psoas muscle morphology and CLBP

Paraspinal muscle degeneration is a complex and dynamic process that may have persistent adverse effects on spinal stability and the local mechanical environment. Because paraspinal muscles are critical for maintaining lumbar segmental stability, controlling abnormal micromotion, and distributing local loads, structural and compositional changes in these muscles have been closely associated with degenerative lumbar disorders, including low back pain and lumbar disc herniation (18).Muscle cross-sectional area (CSA) has been widely used to evaluate paraspinal muscle atrophy. However, whether paraspinal muscle CSA is clearly reduced in patients with CLBP remains controversial. Using quantitative magnetic resonance imaging, Huang et al. (19) reported that paraspinal muscle CSA was not consistently reduced in patients with CLBP compared with healthy controls. Significant differences were detected only in the multifidus muscle at the L4/5 level. In contrast, proton density fat fraction (PDFF) values were significantly increased in the multifidus and erector spinae muscles at the L4/5 and L5/S1 levels. Moreover, PDFF showed stronger correlations than CSA with adjacent intervertebral disc degeneration, ODI, and VAS scores. These findings suggest that paraspinal muscle degeneration in CLBP may be reflected less by muscle size reduction than by changes in muscle composition and quality. Similarly, Barker et al. (20) showed that, in patients with unilateral CLBP, multifidus CSA was lower on the painful side than on the asymptomatic side. Other studies have shown reduced paraspinal muscle strength during maximal isometric trunk flexion and extension in patients with CLBP. However, CSA reductions observed in some studies did not always reach statistical significance (21). One possible explanation is that fatty infiltration and fibrotic replacement may partially fill the space created by muscle atrophy. As a result, total CSA may remain relatively unchanged despite a marked reduction in effective contractile tissue. Therefore, CSA alone may not adequately reflect the extent of paraspinal muscle degeneration in patients with CLBP. In contrast, fat infiltration–related parameters may better reflect reduced muscle quality and functional impairment. These observations are consistent with the findings of the present study. Functional cross-sectional area, which reflects effective contractile muscle tissue, did not differ significantly between the CLBP and non-CLBP groups. In contrast, both MF FI and ES FI were significantly higher and were independently associated with postoperative CLBP in multivariable analyses. These findings indicate that paraspinal muscle degeneration may be characterized less by overt muscle atrophy than by increased fatty replacement, reduced functional muscle components, and impaired stabilizing capacity. Therefore, fat infiltration–related indices may be more sensitive imaging biomarkers than simple area-based measurements for assessing postoperative CLBP risk. The multifidus and erector spinae muscles have distinct but complementary roles in the dynamic stabilization of the spine. The multifidus muscle is located deep in the posterior spine and is primarily responsible for fine segmental control. It plays an important role in limiting abnormal micromotion, maintaining local stability, and regulating intervertebral load distribution (22). In contrast, the erector spinae muscles contribute mainly to trunk extension, postural maintenance, and overall mechanical balance (23). Therefore, increased fatty infiltration in these muscles reflects not only changes in intramuscular composition but also reduced effective contractile tissue and impaired stabilizing function. Under these conditions, mechanical support from the active spinal stabilization system may be weakened. The ability to control abnormal stress and minor displacement during daily activities may also be reduced, increasing susceptibility to localized stress concentration and repetitive low-intensity mechanical stimulation. Paraspinal muscles are important active stabilizers of the lumbar spine and act together with passive structures, including intervertebral discs, ligaments, and facet joints, to maintain spinal stability (24). As fatty infiltration of the multifidus and erector spinae muscles progresses, part of their stabilizing and load-sharing function may be lost. Consequently, spinal stability during standing, walking, and positional changes may become increasingly dependent on passive structures. This local stress concentration may increase the mechanical burden on pain-sensitive structures, including the facet joints, intervertebral discs, and posterior ligaments. It may also expose postoperative tissues to a persistently unfavourable mechanical environment (25).In addition to local paraspinal muscle degeneration, insufficient overall muscle reserve may contribute to postoperative CLBP. In the present study, lower PMI values were significantly associated with a higher risk of postoperative CLBP. This finding suggests that persistent postoperative pain may be related to both impaired local stabilizing muscles and insufficient overall core muscle reserve. PMI, which standardizes psoas muscle CSA by body size, is considered a relatively stable indicator of overall muscle reserve (26). Similarly, Atik et al. (27) reported that reduced psoas-related parameters were significantly associated with osteoporotic vertebral compression fractures. This finding further supports the clinical value of psoas-based sarcopenia assessment in degenerative spinal disorders. The psoas major plays an important role in maintaining lumbar lordosis, trunk stability, and load transfer. Reduced PMI may weaken active spinal stabilization and increase dependence on passive structures, such as intervertebral discs, ligaments, and facet joints, during daily activities. This process may further increase local stress concentration and the risk of persistent pain (28). In addition, reduced PMI may indicate limited potential for postoperative functional recovery and impaired restoration of neuromuscular control, which may further compromise pain relief.

### 4.2 Association between abnormal glucose metabolism and CLBP

Abnormal glucose metabolism is a systemic pathological condition primarily characterized by chronic hyperglycaemia. Its effects are not limited to glucose metabolic disturbance but may also involve the nervous, vascular, immune, and musculoskeletal systems. Consequently, tissue repair, pain regulation, and postoperative functional recovery may be persistently impaired. Previous studies have shown that long-term hyperglycaemia may impair local oxygen and nutrient supply by inducing chronic low-grade inflammation, increasing oxidative stress, and causing microvascular perfusion dysfunction. These changes may delay neural and soft tissue repair (29). A hyperglycaemic environment may also increase nervous system sensitivity to painful stimuli and weaken endogenous pain inhibitory mechanisms. As a result, patients may remain susceptible to persistent or recurrent pain even after neural decompression has been achieved (30).In patients undergoing lumbar decompression surgery, these biological alterations mediated by abnormal glucose metabolism may contribute to discordance between radiographic decompression and clinical pain relief. This discordance may increase the risk of chronic postoperative pain (31). Furthermore, the nonlinear association observed in the present study suggests that the effect of abnormal glucose metabolism on postoperative pain outcomes may not follow a simple linear pattern but may exhibit threshold-related characteristics. When HbA1c levels remain within a relatively controlled range, their influence on postoperative pain outcomes may be limited. However, once metabolic imbalance exceeds a certain threshold, inflammation, microcirculatory dysfunction, and neural sensitization may interact and become amplified, thereby substantially increasing the risk of CLBP. Importantly, abnormal glucose metabolism and skeletal muscle degeneration may not be independent processes (32). Skeletal muscle is essential for maintaining spinal stability and motor function and is also a major site of glucose uptake and energy metabolism. Under chronic hyperglycaemic conditions, accumulation of advanced glycation end products, persistent low-grade inflammation, and microcirculatory dysfunction may collectively promote muscle fatty infiltration, reduce effective contractile tissue, and decrease functional reserve (33, 34). In addition, advanced glycation end product accumulation may alter collagen cross-linking, increase tissue stiffness, and reduce muscle elasticity. These changes may further affect the biomechanical properties of paraspinal soft tissues (35). Chronic hyperglycaemia and insulin resistance may also induce mitochondrial dysfunction, abnormal lipid metabolism, and chronic inflammation in skeletal muscle. These processes may further promote fatty infiltration and muscle atrophy (36).These changes, commonly referred to as diabetic myopathy, may impair paraspinal muscle stabilizing function and reduce overall muscle reserve. In patients with lumbar degenerative disease, these effects may further reduce paraspinal muscle quality and weaken the dynamic spinal stabilization system. Overall muscle reserve and postoperative functional recovery capacity may also be reduced. Consequently, adequate pain relief may remain difficult to achieve even after surgical relief of neural compression.

### 4.3 Association between age and CLBP

This study found that increasing age was independently associated with CLBP after UBE decompression. This suggests that age is not only a demographic characteristic but may also represent a biological factor influencing postoperative pain outcomes. Previous studies have shown that spinal degenerative changes accumulate with age. Structural changes, including disc dehydration, endplate degeneration, facet joint hypertrophy, and reduced ligament elasticity, become more common with age. These changes may alter the mechanical environment of the spine (37). Older patients are also more likely to show increased paraspinal muscle fat infiltration, reduced muscle quality, and decreased overall muscle reserve. These factors may further impair local dynamic stability. In patients undergoing lumbar decompression, neural compression may be adequately relieved after surgery. However, when substantial age-related degeneration is present preoperatively, postoperative tissues may remain vulnerable to persistent pain. This vulnerability may be related to reduced stability, impaired load regulation, and diminished repair capacity. Beyond structural degeneration, ageing may contribute to postoperative CLBP through impaired tissue repair and altered neural regulation. With increasing age, microcirculatory function, metabolic reserve, and tissue regenerative capacity may decline. As a result, resolution of postoperative inflammation, soft tissue healing, and neural recovery may be delayed (38). Older patients may also have reduced pain modulation capacity and a higher burden of chronic comorbidities, which may further increase the risk of persistent pain. Therefore, the effect of age on postoperative CLBP may reflect more than slower recovery alone. It may represent the combined effects of accumulated degeneration, reduced muscle function, and impaired repair capacity. Notably, RCS analysis showed a nonlinear association between age and postoperative CLBP risk. The increase in risk was more pronounced in older patients. The curve suggested that, between approximately 40 and 70 years of age, the overall risk remained relatively stable, without a clear upward trend. After approximately 70 years of age, the risk began to increase gradually. A steeper increase was observed after 75 years of age, with a more rapid rise beyond 80 years. These findings suggest that the effect of age on postoperative pain outcomes may not be uniform and may involve a threshold or acceleration pattern. At younger ages, compensatory mechanisms and functional recovery capacity may be relatively preserved; therefore, the impact of age on pain risk may be limited. At more advanced ages, multiple adverse factors, including spinal degeneration, reduced muscle quality, delayed tissue repair, and impaired pain regulation, may interact and lead to a more pronounced increase in CLBP risk. It should be noted that the confidence intervals were wider in the older age range, indicating some uncertainty in risk estimation. However, the overall trend still supports an increased risk of postoperative CLBP in older patients. These findings suggest that age should not be treated only as a linear adjustment variable in preoperative risk assessment. Instead, its potential nonlinear effects should be considered, and more refined risk stratification and perioperative management may be needed for older patients.

### 4.4 Limitations

This study has several limitations. First, this was a multicentre retrospective study. Although uniform inclusion and exclusion criteria, outcome definitions, and surgical procedures were applied across centres, and multiple confounders were adjusted for in the statistical analyses, selection and information biases could not be completely avoided because of the retrospective design. Some potential confounders may not have been fully controlled. Therefore, the present findings should be interpreted as associations rather than direct causal relationships. Second, all included patients had single-level lumbar disc herniation and underwent UBE decompression. Thus, the conclusions are primarily applicable to this specific population. Caution is warranted when extrapolating these findings to patients with multilevel disease, marked instability requiring fusion, or other types of degenerative lumbar disorders. Third, paraspinal and psoas muscle assessments were based mainly on preoperative imaging at a single time point. Therefore, dynamic changes in muscle degeneration and recovery during the perioperative period could not be evaluated. In addition, paraspinal muscle measurements were obtained only at the L4 lower endplate level. Although this approach improved measurement consistency, it may not fully represent the overall condition of paraspinal muscles at different spinal levels. Fourth, although HbA1c provides a relatively stable measure of glycaemic control over a certain period, diabetes duration, glucose-lowering regimens, perioperative glucose fluctuations, and other metabolic indicators were not included in this study. Therefore, the dynamic effects of abnormal glucose metabolism on postoperative CLBP could not be fully clarified. Fifth, although patients with long-term preoperative analgesic use were excluded, analgesic use during follow-up was not systematically recorded or included in the statistical analyses. In addition, postoperative rehabilitation adherence, diabetes duration, glucose-lowering strategies, inflammatory markers, neurophysiological findings, and muscle strength measurements were not analysed. Therefore, the potential mechanisms linking glucose metabolic status, paraspinal muscle fatty infiltration, PMI, and postoperative CLBP could not be fully verified. Future prospective studies with multidimensional follow-up are needed. Functional and biological indicators should also be incorporated to further clarify the pathways linking abnormal glucose metabolism, muscle degeneration, and chronic postoperative pain.

## 5 Conclusion

The risk of CLBP after endoscopic lumbar decompression was associated not only with local decompression but also with preoperative glucose metabolism status, paraspinal muscle fat infiltration, and overall muscle reserve. Metabolic abnormalities, degeneration of the local stabilising system, and reduced muscle reserve may collectively contribute to persistent postoperative pain.

## Data Availability

The raw data supporting the conclusions of this article will be made available by the authors, without undue reservation.
